# Fucoxanthin prevents H_2_O_2_-induced neuronal apoptosis via concurrently activating the PI3-K/Akt cascade and inhibiting the ERK pathway

**DOI:** 10.1080/16546628.2017.1304678

**Published:** 2017-03-28

**Authors:** Jie Yu, Jia-Jia Lin, Rui Yu, Shan He, Qin-Wen Wang, Wei Cui, Jin-Rong Zhang

**Affiliations:** ^a^ Ningbo Key Laboratory of Behavioral Neuroscience, Department of Physiology, School of Medicine, Ningbo University, Ningbo, PRChina; ^b^ School of Marine Sciences, Ningbo University, Ningbo, Ningbo, PRChina

**Keywords:** Fucoxanthin, neurodegenerative disorders, H_2_O_2_, Akt, ERK

## Abstract

**Background**: As a natural carotenoid abundant in chloroplasts of edible brown algae, fucoxanthin possesses various health benefits, including anti-oxidative activity in particular.

**Objective**: In the present study, we studied whether fucoxanthin protected against hydrogen peroxide (H_2_O_2_)-induced neuronal apoptosis.

**Design**: The neuroprotective effects of fucoxanthin on H_2_O_2_-induced toxicity were studied in both SH-SY5Y cells and primary cerebellar granule neurons.

**Results**: Fucoxanthin significantly protected against H_2_O_2_-induced neuronal apoptosis and intracellular reactive oxygen species. H_2_O_2_ treatment led to the reduced activity of phosphoinositide 3-kinase (PI3-K)/Akt cascade and the increased activity of extracellular signal-regulated kinase (ERK) pathway in SH-SY5Y cells. Moreover, fucoxanthin significantly restored the altered activities of PI3-K/Akt and ERK pathways induced by H_2_O_2_. Both specific inhibitors of glycogen synthase kinase 3β (GSK3β) and mitogen-activated protein kinase kinase (MEK) significantly protected against H_2_O_2_-induced neuronal death. Furthermore, the neuroprotective effects of fucoxanthin against H_2_O_2_-induced neuronal death were abolished by specific PI3-K inhibitors.

**Conclusions**: Our data strongly revealed that fucoxanthin protected against H_2_O_2_-induced neurotoxicity via concurrently activating the PI3-K/Akt cascade and inhibiting the ERK pathway, providing support for the use of fucoxanthin to treat neurodegenerative disorders induced by oxidative stress.

## Introduction

Oxidative stress plays a critical role in neuronal loss of neurodegenerative disorders.[1] Excessive reactive oxygen species (ROS), including hydrogen peroxide (H_2_O_2_), nitric oxide and highly reactive hydroxyl radicals, are released after the neuron injury during oxidative stress. ROS further promote neurotoxicity via interacting with macromolecules and regulating signaling pathways.[2] Among various ROS, H_2_O_2_ is widely used to establish oxidative stress-induced neurotoxicity model *in vitro*.[3] H_2_O_2_-induced neurotoxicity is regulated by the inhibition of pro-survival pathways, such as the phosphoinositide 3-kinase (PI3-K)/Akt cascade, and/or the activation of pro-apoptotic pathways, such as the mitogen-activated protein kinase (MAPK) pathway. Inhibition of PI3-K/Akt cascade consequently activates glycogen synthase kinase 3β (GSK3β), a molecule involved in H_2_O_2_-induced neuronal apoptosis.[4,5] In addition, extracellular signal-regulated kinase (ERK), a key intermediate of the MAPK pathway, has been regarded as a main pro-apoptotic molecule involved in H_2_O_2_-induced neurotoxicity.[5,6]

Fucoxanthin is one of the most abundant marine carotenoids found in edible brown seaweeds.[7] Previous investigations have reported that fucoxanthin possesses different health benefits, including anti-oxidative activity in particular.[8–10] Fucoxanthin significantly reduces weight gain in animals through enhancing fatty acid oxidation in white adipose tissue.[11] Moreover, fucoxanthin-fed animals display reduced levels of oxidative stress markers, as well as enhanced activities of antioxidant enzymes.[12] Fucoxanthin has also been reported to produce anti-oxidative property in various *in vitro* models, including Aβ_42_-treated microglia cells, ferric nitrilotriacetate-treated hepatic cells and UV-induced fibroblast cells.[13–16] However, it remains unclear whether fucoxanthin could protect neuronal cells against oxidative stress-related neurotoxicity.

As human neuroblastoma, SH-SY5Y cells are sensitive to oxidants.[17] Therefore, SH-SY5Y cells are used as cellular models to explore the molecular mechanisms of oxidative stress-induced neurotoxicity.[18,19] Moreover, homogenous cerebellar granule neurons (CGNs) can be easily obtained because more than 90% of cells in cerebellum are CGNs.[20] Therefore, CGNs are widely used as a model of primary neurons to examine neuroprotective chemicals.

In our study, we showed that fucoxanthin effectively protected against H_2_O_2_-induced neurotoxicity in both SH-SY5Y cells and primary CGNs. Our results also demonstrated that fucoxanthin exerted such neuroprotective effects via concurrently activating the PI3-K/Akt cascade and inhibiting the ERK pathway.

## Materials and methods

### Chemicals and reagents

Fucoxanthin was extracted from *Sargussum horneri* using a series of steps, including solvent extraction, ethanol precipitation and low-temperature concentration. Briefly, fucoxanthin isolation was conducted at 30°C for 2 h with ethanol to sample ratio of 4:1 (v/w). Then the fucoxanthin-containing solution was concentrated at 25°C. Lipid and chlorophylls were precipitated when the ethanol content reached 63% in the concentrated solution. Fucoxanthin was purified by precipitation when the ethanol content reached 40% in the solution. The purity of fucoxanthin was more than 90% by HPLC, and purified fucoxanthin was stored at −20°C prior to further analysis. H_2_O_2_ was purchased from Calbiochem (San Diego, CA, USA). SB415286 was purchased from Sigma Chemicals (St Louis, MO, USA). U0126, Wortmannin and LY294002 were supplied from LC Laboratories (Woburn, MA, USA). Antibodies against pSer473-Akt, Akt, pSer9-GSK3β, GSK3β, pERK and ERK were provided by Cell Signaling Technology (Beverly, MA, USA). Unless otherwise noted, all media and supplements for cell cultures were obtained from Invitrogen (Carlsbad, CA, USA).

### Culture of SH-SY5Y cells

SH-SY5Y cells were purchased from the Shanghai Institute of Cell Biology (Chinese Academy of Sciences, Shanghai, China) and maintained in high glucose modified Eagle’s medium (DMEM) supplemented with 10% fetal bovine serum (FBS) and penicillin (100 U^ ^ml^–1^)/streptomycin (100 μg^ ^ml^–1^) at 37°C with 5% CO_2_ in a humidified environment. The medium was refreshed every other day. For the H_2_O_2_ experiment, SH-SY5Y cells in DMEM with low serum content (1% FBS) were seeded in six-well or 96-well plates at a density of 1 × 10^5^ cells^ ^ml^–1^ for 24 h before further experiments.

### Culture of primary CGNs

CGNs were isolated from eight-day-old Sprague-Dawley rats as previously described.[21] Briefly, cells were seeded at a density of 2.7 × 10^5^ cells cm^–^^2^ and maintained in basal modified Eagle’s medium supplemented with 10% FBS, 25 mM KCl, 2 mM glutamine and penicillin (100 U^ ^ml^–1^)/streptomycin (100 μg^ ^ml^–1^) for 24 h. Subsequently, cytosine arabinoside (10 μM) was added to the medium to inhibit the growth of non-neuronal cells. Granule cells were identified according to several criteria, including their size, shape and relative proportion of the total cell population.

### Measurement of cell viability

Cell viability was assessed by the 3(4,5-dimethylthiazol-2-yl)-2.5-diphenyltetrazolium bromide (MTT) assay based on previous protocol.[22,23] Briefly, 10 μl of MTT solution (5 mg^ ^ml^–1^) was added to each well after treatment. Plates were incubated at 37°C for 4 h in a humidified incubator. Subsequently, 100 μl of the solvating solution (0.01 N HCl in 10% SDS solution) was then added to each well, followed by incubation for 16–20 h. The absorbance of the samples was determined at a wavelength of 570 nm with 655 nm as a reference wavelength. Unless otherwise indicated, the extent of MTT conversion in cells was expressed as a percentage of the control without treatment.

### Fluorescein diacetate/propidium iodide double staining assay

Viable cells were visualized by the fluorescein formed from fluorescein diacetate (FDA) by esterase activity in viable cells. Non-viable cells were analyzed by propidium iodide (PI) staining, which only penetrates the membranes of dead cells. Briefly, the cells were examined after incubation with 10 μg^ ^ml^–1^ of FDA and 5 μg^ ^ml^–1^ of PI for 15 min. Images were acquired using UV light microscopy and compared with those taken under phase-contrast microscopy. To quantitatively evaluate cell viability, images of each well were taken from five randomly selected fields, and the number of PI-positive cells and FDA-positive cells was counted. The number of FDA-positive cells was then averaged using the equation as follows: % of cell viability = [number of FDA-positive cells /(number of PI-positive cells + number of FDA-positive cells)] × 100%.[24]

### Hoechst staining

Chromatin condensation was evaluated by staining the cell nuclei with Hoechst 33,342 as previously described.[25,26] After treatment, cells were washed with ice-cold phosphate buffered saline (PBS), fixed with 4% formaldehyde for 15 min, membrane-permeabilized in 0.1% Triton X-100 for 15 min, and blocked in 1% bovine serum albumin (BSA) for 15 min. Cells were then stained with Hoechst 33,342 (5 μg^ ^ml^–1^) at 4°C for 5 min. Images were obtained using a fluorescence microscope (Nikon Instruments Inc., Melville, NY, USA) at 100× magnification. Ultraviolet excitation and emission wavelengths were used to obtain images of nuclei labeled with Hoechst-33342. To determine the proportion of apoptotic nuclei in each group, images of each well were taken from five randomly selected fields, and the number of pyknotic nuclei and total nuclei was counted. The percentage of pyknotic nuclei was then averaged.

### Measurement of intracellular ROS

Intracellular ROS was measured by 2′7′-dichlorodihydrofluorescein diacetate (DCFH-DA, Molecular Probes, Eugene, OR, USA), a fluorescent dye which could be converted to membrane impermeable derivative DCFH.[27] In the presence of intracellular ROS, DCFH is oxidized to highly fluorescent 2′,7′-dichlorofluorescein (DCF). Cells were washed with PBS and incubated with 10 μM DCFH-DA at 37°C for 15 min. The dye was removed, and cells were washed with PBS and scanned with a plate reader (Wallac, PerkinElmer, Waltham, MA, USA) at 485 nm excitation and 520 nm emission. Images were acquired using a fluorescence microscope (Nikon Instruments Inc.).

### Western blot analysis

Western blotting was performed using a well-established protocol.[28] Cell lysates were separated on SDS–polyacrylamide gels and electrotransferred onto polyvinyldifluoride membranes. After membrane blocking, proteins were detected using primary antibodies. After incubation at 4°C overnight, signals were obtained after incubation with HRP-conjugated secondary antibodies (Santa Cruz Biotechnology, Santa Cruz, CA, USA). Subsequently, blots were developed using the enhanced chemiluminescence plus kit (Amersham Bioscience, Aylesbury, UK) and signals were exposed to autoradiographic film.

### Statistical analysis

Results were expressed as mean ± SEM. Differences among groups were compared by analysis of variance (ANOVA) followed by Dunnett’s or Tukey’s test. *P* < 0.05 was considered as statistically significant.

## Results

### Fucoxanthin reduces H_2_O_2_-induced neuronal apoptosis in SH-SY5Y cells

**Figure 1.**
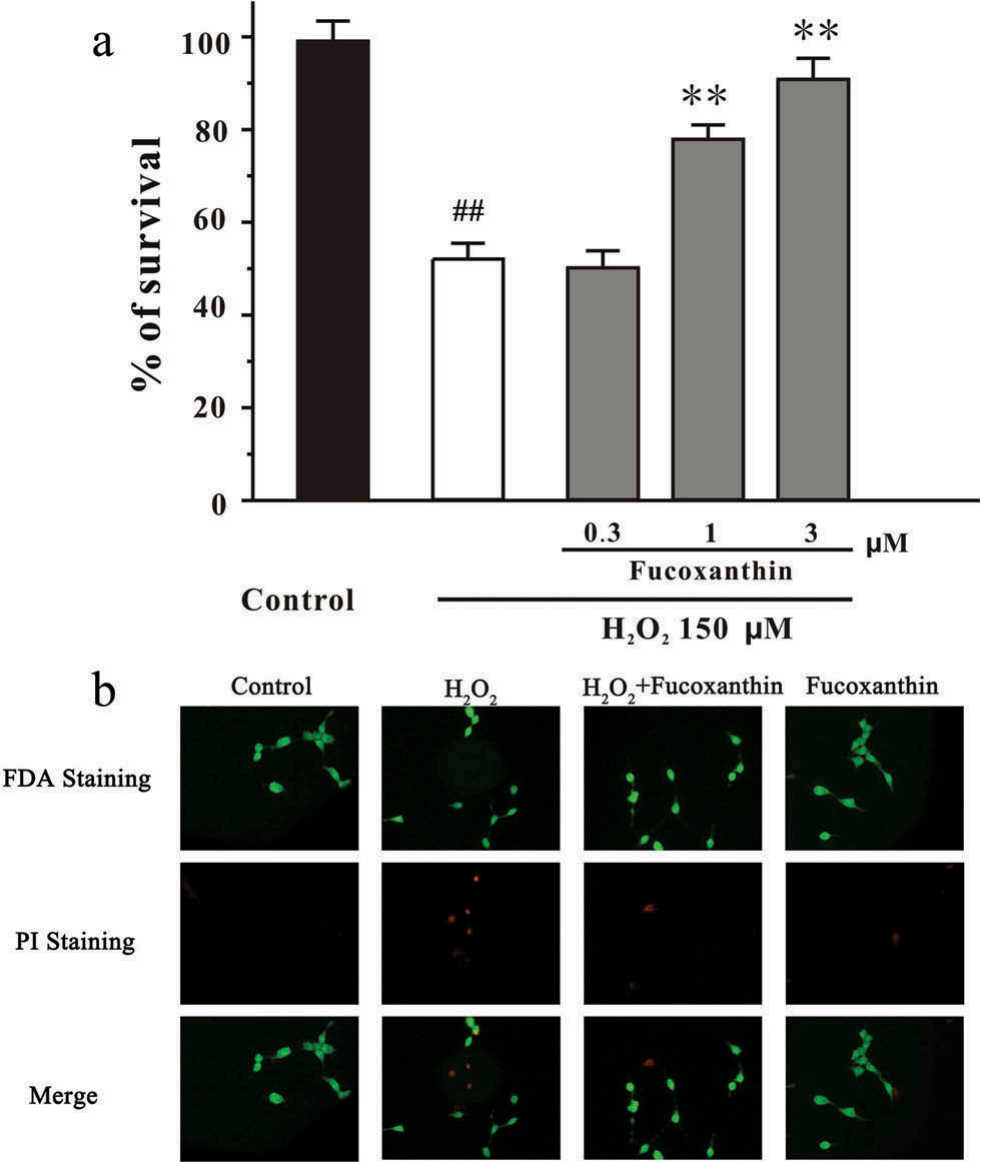
Fucoxanthin protects against H_2_O_2_-induced cell death in a dose-dependent manner. (a) SH-SY5Y cells were treated with fucoxanthin. After 2 h, cells were exposed to 150 μM H_2_O_2_. MTT assay was used to measure cell viability at the 24 h after H_2_O_2_ exposure. Data, expressed as the percentage of control, were presented as the mean ± SEM of three separate experiments; ^##^ *p *< 0.01 vs. the control group, **p < 0.01 vs. the H_2_O_2_-challenged group (ANOVA and Tukey’s test). (b) SH-SY5Y cells were administrated with 3 μM fucoxanthin for 2 h, and exposed to 150 μM H_2_O_2_. After 24 h, cells were examined by FDA/PI double staining.

**Figure 2.**
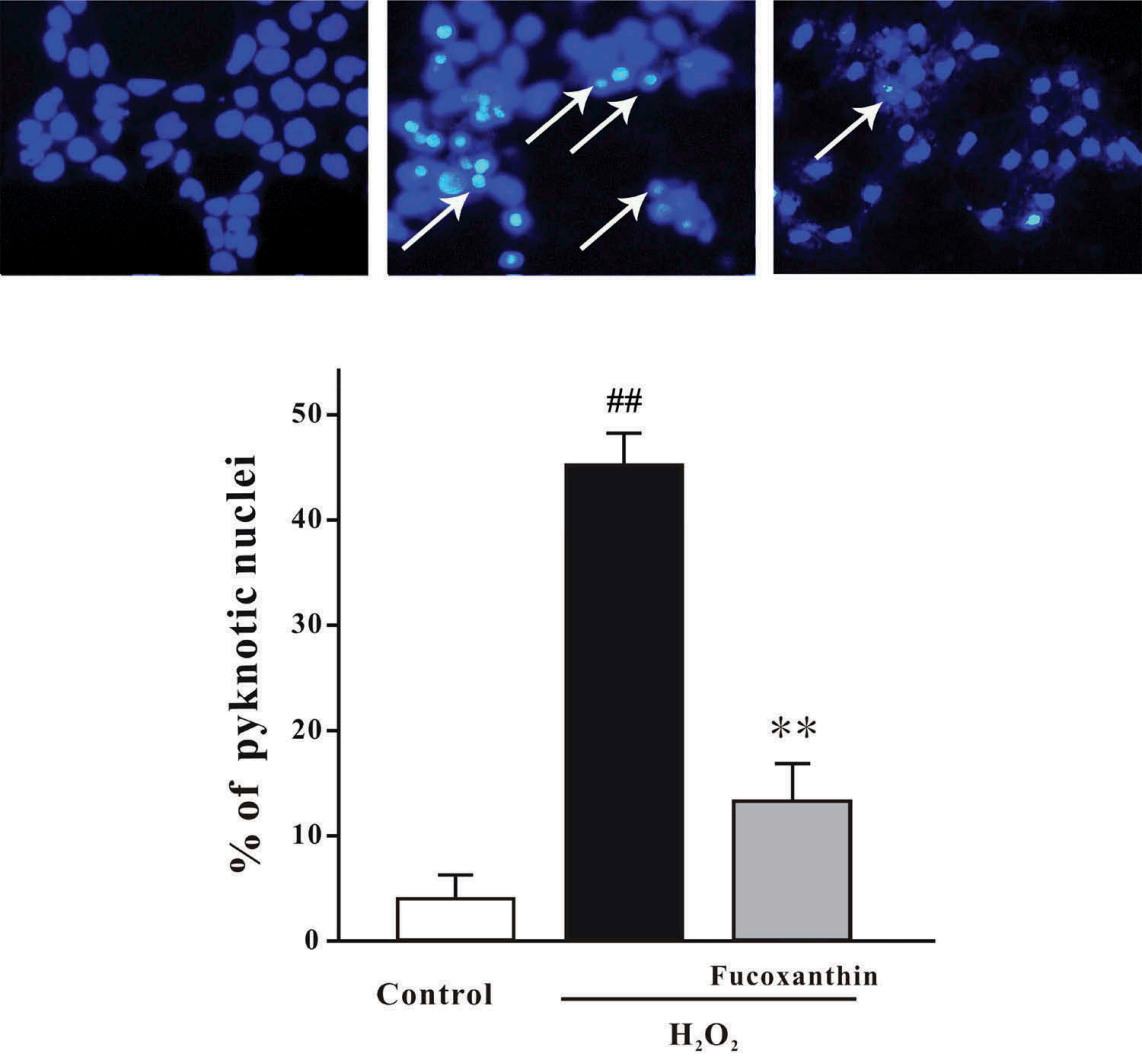
Fucoxanthin significantly protects against H_2_O_2_-induced apoptosis in SH-SY5Y cells. SH-SY5Y cells were treated with 3 μM fucoxanthin. After 2 h, cells were exposed to 150 μM H_2_O_2_. Cells were measured by Hoechst staining at 24 hours after H_2_O_2_ challenge. The number of pyknotic nuclei was counted. Data were presented as the mean ± SEM of three separate experiments; ^##^ *p *< 0.01 vs. the control group, **p < 0.01 vs. the H_2_O_2_- challenged group (ANOVA and Tukey’s test). Array: representative pyknotic nuclei.

In the present study, SH-SY5Y cells were first pre-treated with fucoxanthin ranging from 0.3 to 3 μM for 2 h, followed by treatment with 150 μM H_2_O_2_ for 24 h. Cell viability was determined by the MTT assay. Figure 1(a) shows that fucoxanthin significantly reduced H_2_O_2_-induced neuronal death in a dose-dependent manner. The cell viability in H_2_O_2_, 1 μM fucoxanthin + H_2_O_2_ and 3 μM fucoxanthin + H_2_O_2_ groups were 52.4, 79.0 and 98.1%, respectively. Treatment with 3 μM fucoxanthin alone for 26 h was not cytotoxic and did not alter cell proliferation (data not shown). To further elucidate the neuroprotective effect of fucoxanthin against H_2_O_2_-induced neurotoxicity, SH-SY5Y cells were analyzed by FDA/PI double staining. Figure 1(b) exhibits that fucoxanthin blocked the loss of neurons induced by H_2_O_2_. Moreover, fucoxanthin significantly decreased H_2_O_2_-induced neuronal apoptosis in SH-SY5Y cells (Figure 2).

### Fucoxanthin reduces H_2_O_2_-induced neuronal death in primary CGNs

**Figure 3.**
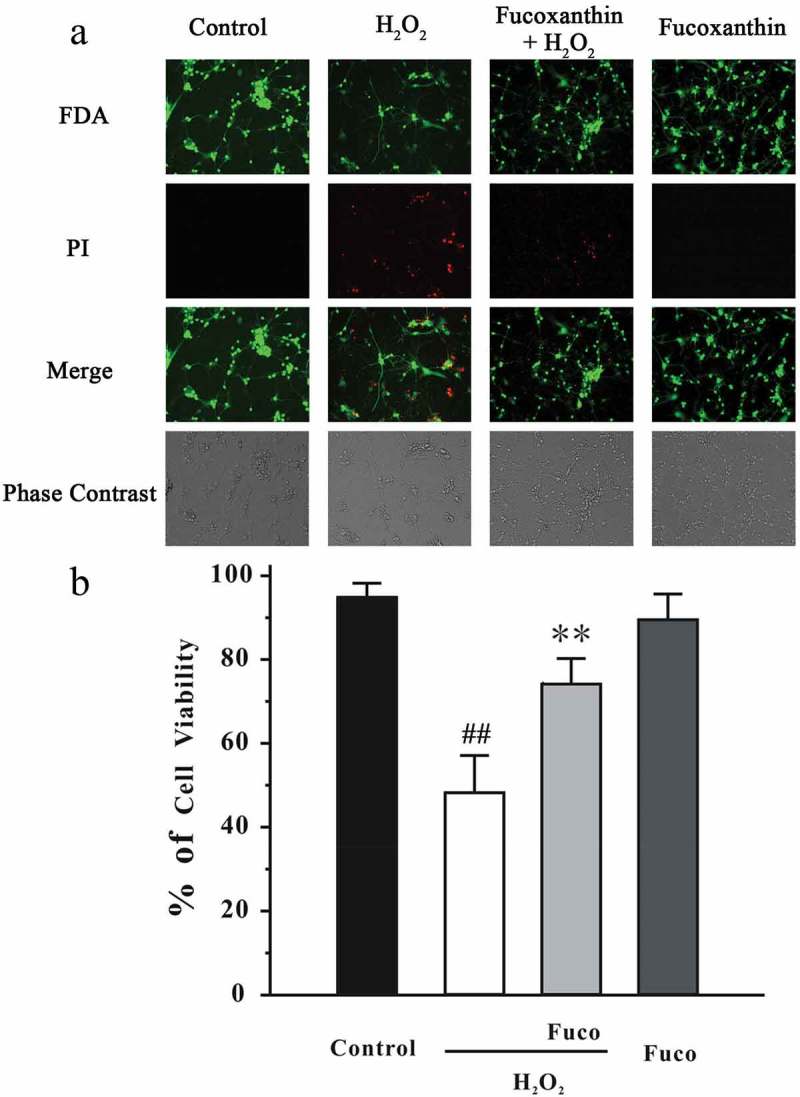
Fucoxanthin reduces H_2_O_2_-induced cell death in primary CGNs. (a) CGNs were administrated with 3 μM fucoxanthin (Fuco). After 2 h, cells were exposed to 30 μM H_2_O_2_. At 6 h after H_2_O_2_ exposure, CGNs were analyzed by FDA/PI staining. Cell viability was analyzed from representative photomicrographs. Data were presented as the mean ± SEM of three separate experiments; ^##^ *p *< 0.01 vs. the control group, **p < 0.01 vs. the H_2_O_2_-challenged group (ANOVA and Tukey’s test).

We have previously demonstrated that the treatment of 30 μM H_2_O_2_ for 6 h leads to significant neuronal death in CGNs.[29] Therefore, we further investigated whether fucoxanthin could produce neuroprotective effects in CGNs. CGNs were pre-treated with fucoxanthin for 2 h, and then treated with 30 μM H_2_O_2_ for another 6 h. CGNs were examined by FDA/PI double staining. Figure 3 displays that treatment of 3 μM fucoxanthin significantly reduced H_2_O_2_-induced neuronal death in CGNs. The cell viability in H_2_O_2_ and 3 μM fucoxanthin + H_2_O_2_ groups were 48.8% and 73.4%, respectively. Treatment with 3 μM fucoxanthin alone for 26 h did not alter cell viability in CGNs (data not shown).

### Fucoxanthin reduces H_2_O_2_-induced intracellular ROS in SH-SY5Y cells

**Figure 4.**
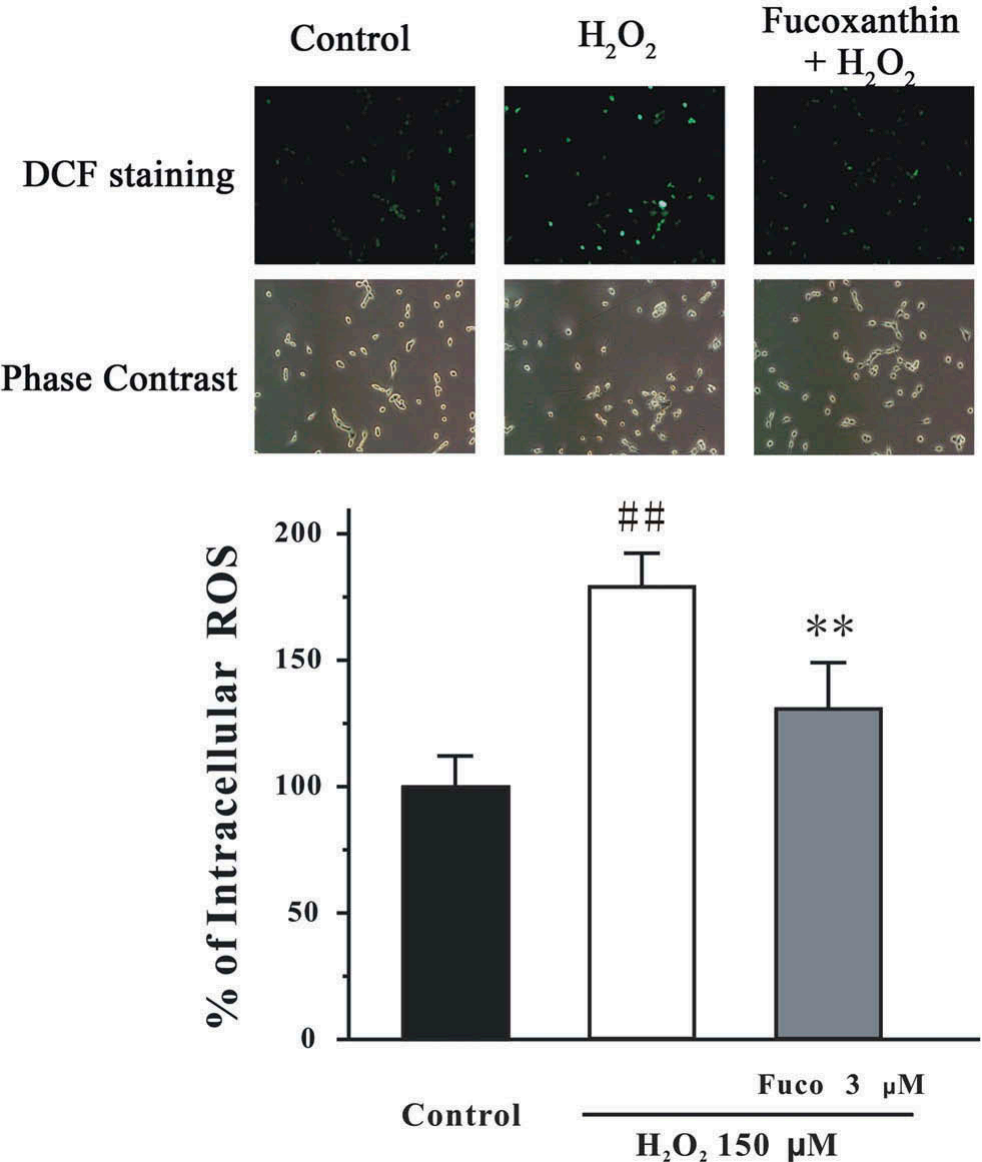
Fucoxanthin significantly protected against H_2_O_2_-induced increase of intracellular ROS in SH-SY5Y cells. SH-SY5Y cells were treated with 3 μM fucoxanthin. After 2 h, cells were exposed to 150 μM H_2_O_2_. Intracellular ROS was measured by DCF-DA assay at 2 h after H_2_O_2_ challenge. Data were presented as the mean ± SEM of three separate experiments; ^##^ *p *< 0.01 vs. the control group, **p < 0.01 vs. the H_2_O_2_-challenged group (ANOVA and Tukey’s test).

We have also measured intracellular ROS by using DCF-DA. We found that H_2_O_2_ significantly increased intracellular ROS in SH-SY5Y cells (Figure 4). Moreover, fucoxanthin significantly reversed the increased intracellular ROS induced by H_2_O_2_. These results suggested that H_2_O_2_ could induce oxidative stress which could be decreased by fucoxanthin.

### Inhibiting the PI3-K/Akt cascade and activating the ERK signaling are involved in H_2_O_2_-induced neurotoxicity in SH-SY5Y cells

**Figure 5.**
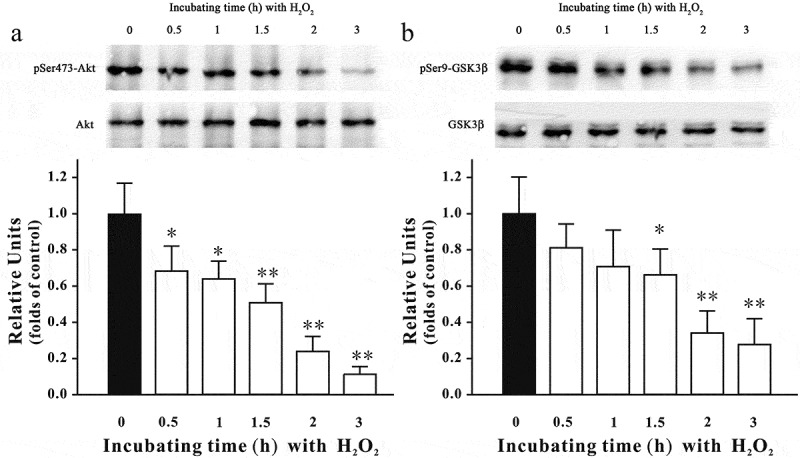
H_2_O_2_ decreases pSer473-Akt and pSer9-GSK3β in a time-dependent manner. SH-SY5Y cells were treated with 150 μM H_2_O_2_ for various durations as indicated. Western blot analysis was used to assess the expression of (a) pSer473-Akt and (b) pSer9-GSK3β. Data were presented as the mean ± SEM of three separate experiments; *p < 0.05 and **p < 0.01 vs. control group (ANOVA and Dunnett’s test).

**Figure 6.**
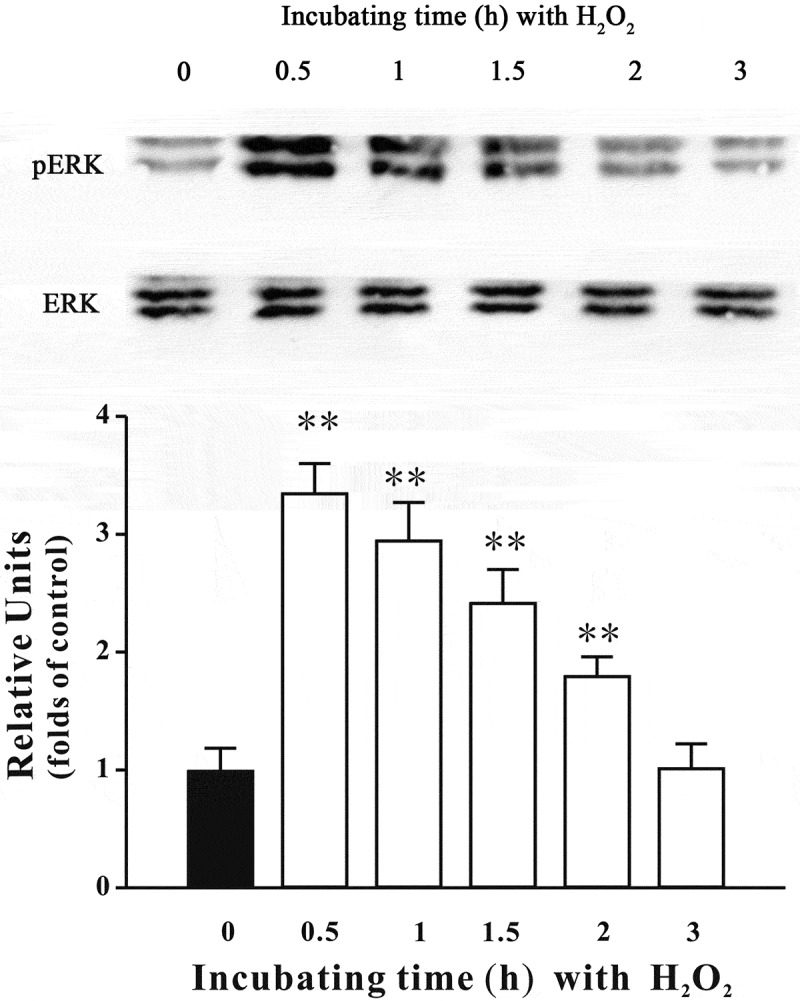
H_2_O_2_ increases the level of pERK in SH-SY5Y cells. SH-SY5Y cells were treated with 150 μM H_2_O_2_ for various durations as indicated. Western blot was used to analyze pERK. Data were presented as the mean ± SEM of three separate experiments; **p < 0.01 vs. control group (ANOVA and Dunnett’s test).

**Figure 7.**
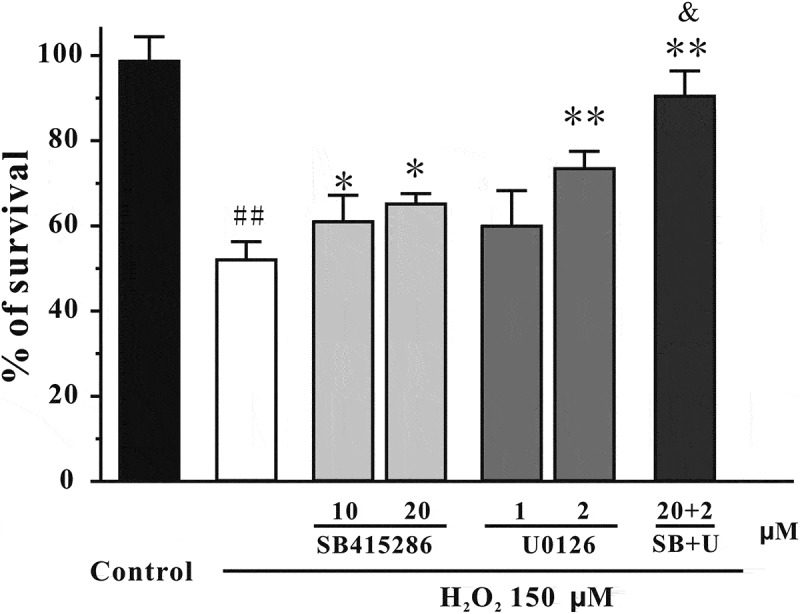
GSK3β and MEK specific inhibitors protect against H_2_O_2_-induced cell death. SH-SY5Y cells were treated with SB415286, U0126 or SB415286 + U0126 (SB+U) at the indicated concentrations. After 2 h, cells were exposed to 150 μM H_2_O_2_. Cell viability was analyzed by MTT assay at 24 h after H_2_O_2_ exposure. Data were presented as the mean ± SEM of three separate experiments; ^##^ *p *< 0.01 vs. the control group, *p < 0.05 and **p < 0.01 vs. H_2_O_2_-treated group, ^&^ *p *< 0.05 vs. H_2_O_2_ + 20 μM SB415286 group (ANOVA and Tukey’s test).

Previous studies have reported that inhibition of the PI3-K/Akt cascade and activation of the ERK signaling are involved in the neurotoxicity induced by oxidative stress.[4,5] Therefore, we assessed whether the alteration of these pathways was also involved in our model. Figure 5 shows that H_2_O_2_ decreased pSer473-Akt and pSer9-GSK3β levels in SH-SY5Y cells. Moreover, the level of pERK was significantly increased during first 30 min post-treatment, although it returned to its basal level 3 h after H_2_O_2_ challenge (Figure 6). Furthermore, SB415286, a specific inhibitor of GSK3β, and U0126, a specific inhibitor of mitogen-activated protein kinase kinase (MEK), significantly protected against H_2_O_2_-induced neuronal death (Figure 7). Interestingly, co-application of SB415286 and U0126 significantly produced the neuroprotective effects, which were similar to those of fucoxanthin. Taken together, these results suggested that inhibition of the PI3-K/Akt cascade and activation of the ERK signaling were involved in the H_2_O_2_-induced neurotoxicity in SH-SY5Y cells. In addition, fucoxanthin might protect against H_2_O_2_-induced neurotoxicity via regulating these signaling pathways.

### Fucoxanthin protects against the inhibition of the PI3-K/Akt cascade induced by H_2_O_2_

**Figure 8.**
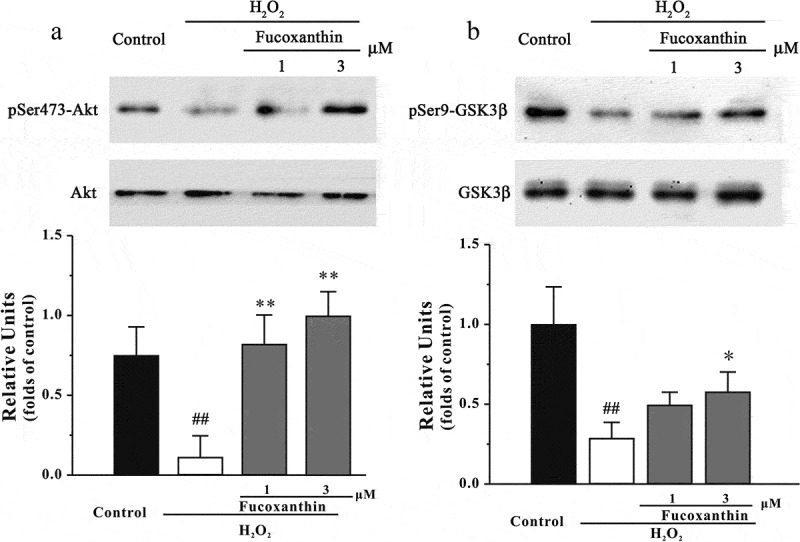
Fucoxanthin protects against H_2_O_2_-induced decrease of pSer473-Akt and pSer9-GSK3β. SH-SY5Y cells were treated with fucoxanthin. After 2 h, cells were exposed to 150 μM H_2_O_2_. Western blot was used to analyze (a) pSer473-Akt and (b) pSer9-GSK3β at 3 h after H_2_O_2_ treatment. Data were presented as the mean ± SEM of three separate experiments; ^##^ *p *< 0.01 vs. the control group, *p < 0.05 and **p < 0.01 vs. H_2_O_2_-treated group (ANOVA and Tukey’s test).

**Figure 9.**
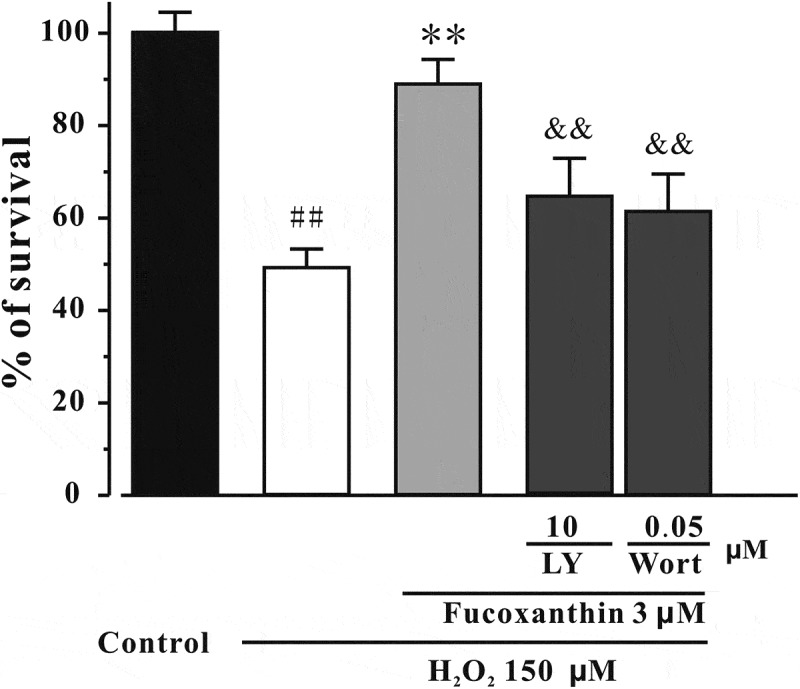
PI3-K specific inhibitors abolish the neuroprotective effects of fucoxanthin. SH-SY5Y cells were pre-treated with LY294002 (LY) or wortmannin (Wort) s for 0.5 h, and then treated with 3 μM fucoxanthin for 4 h before the exposure to 150 μM H_2_O_2_. MTT assay was used to measure cell viability at 24 h after H_2_O_2_ treatment. Data were presented as the mean ± SEM of three separate experiments; ^##^ *p *< 0.01 vs. the control group, **p < 0.01 vs. H_2_O_2_-treated group, ^&&^ *p *< 0.01 vs. H_2_O_2_ plus fucoxanthin group (ANOVA and Tukey’s test).

To examine whether fucoxanthin produced neuroprotective effects via the modulation of the PI3-K/Akt cascade, we assessed the levels of pSer473-Akt and pSer9-GSK3β. Figure 8 shows that pre-treatment with 3 μM fucoxanthin significantly reversed the decrease of both pSer473-Akt and pSer9-GSK3β induced by H_2_O_2_). Additionally, LY294002 and wortmannin, two PI3-K specific inhibitors, were used. Figure 9 shows that the inhibition of PI3-K by either LY294002 or wortmannin significantly abolished the neuroprotective effects of fucoxanthin, suggesting that fucoxanthin protected against H_2_O_2_-induced neuronal death via reversing the inhibition of PI3-K/Akt cascade.

### Fucoxanthin reduces the activation of ERK signaling induced by H_2_O_2_

**Figure 10.**
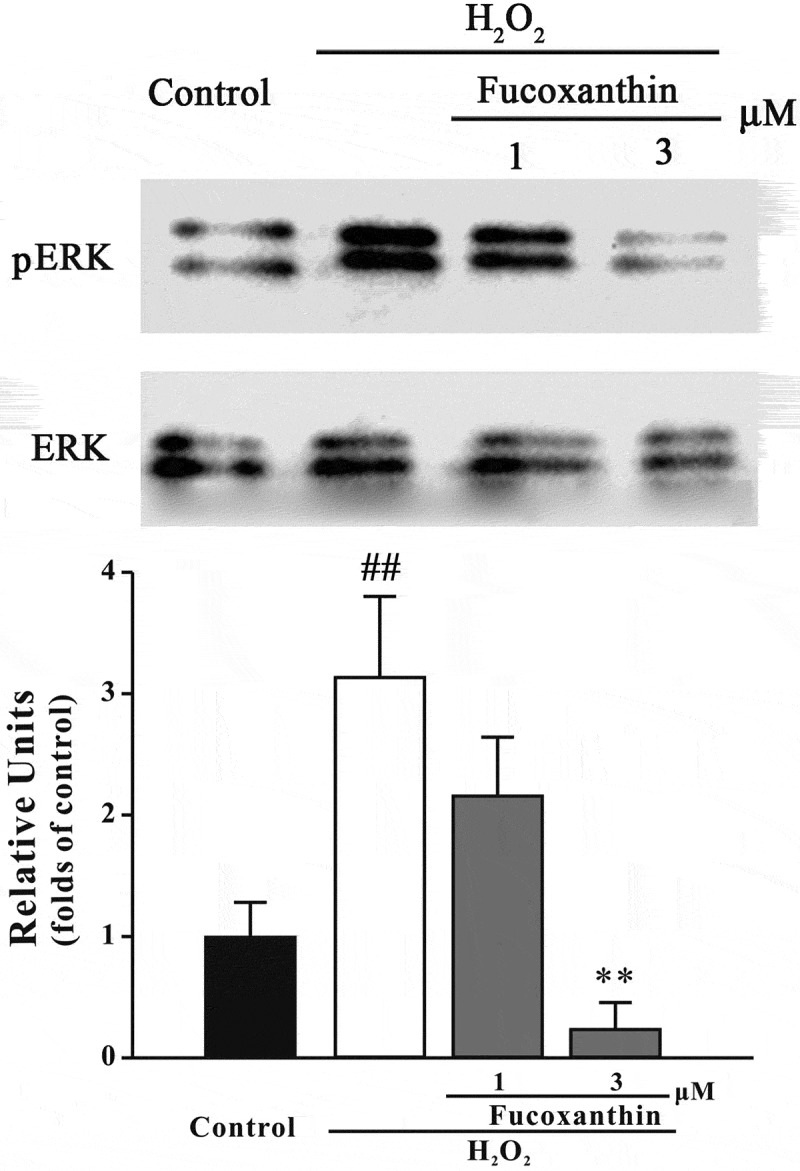
Fucoxanthin inhibits H_2_O_2_-induced increase of pERK level. SH-SY5Y cells were treated with fucoxanthin for 2 h, and then exposed to 150 μM H_2_O_2_. Western blot was used to detect pERK at 30 min after H_2_O_2_ treatment. Data were presented as the mean ± SEM of three separate experiments; ^##^ *p *< 0.01 vs. the control group, **p < 0.01 vs. H_2_O_2_-treated group (ANOVA and Tukey’s test).

In our current study, we examined the level of pERK to further assess whether fucoxanthin produced neuroprotective effects via the inhibition of the ERK pathway. Figure 10 displays that fucoxanthin pre-treatment significantly protected against the increase of pERK induced by H_2_O_2_ at 0.5 h, suggesting that fucoxanthin also inhibited the ERK pathway activation.

## Discussion

In our study, we showed that fucoxanthin, a marine carotenoid, potently protected against H_2_O_2_-induced neurotoxicity in SH-SY5Y cells and in primary CGNs. In addition, we found that the neuroprotective effects of fucoxanthin were concurrently mediated via the activation of the PI3-K/Akt cascade and inhibition of the ERK pathway.

Fucoxanthin has been shown to possess different health benefits, such as anti-obesity, anti-tumor, anti-inflammatory as well as hepatoprotective activities.[30] Therefore, fucoxanthin might be used as a drug or a functional food to treat chronic diseases. We have recently reported that fucoxanthin can inhibit acetylcholinesterase and attenuate scopolamine-induced cognitive impairments in mice, indicating that this chemical can be used in the treatment of Alzheimer’s disease.[31] However, it remains unclear whether fucoxanthin could produce neuroprotective effects. Recent studies have reported that fucoxanthin can prevent oxidative stress-induced cytotoxicity in microglia cells, hepatic cells and fibroblast cells.[13–16] Therefore, we first evaluated whether fucoxanthin protected against oxidative stress-induced neurotoxicity. Our data demonstrated that fucoxanthin protected against H_2_O_2_-induced neuronal death not only in SH-SY5Y cells, but also in primary CGNs, providing a strong support that fucoxanthin could produce neuroprotective effects, and it might be used in the treatment of neurodegenerative disorders.

How could fucoxanthin produce neuroprotective effects? Fucoxanthin belongs to the carotenoid group, which are potent antioxidants.[32] Fucoxanthin could quench singlet oxygen and scavenge free radicals, and therefore protect against cancer and inflammatory diseases.[33–35] In our study, we found that fucoxanthin could reduce H_2_O_2_-induced increase of intracellular ROS, suggesting that fucoxanthin, like other carotenoids, exerts its neuroprotective effects via the inhibition of oxidative stress. Previous studies have shown that fucoxanthin could act on many proteins, including the Bcl2 protein family, MAPK, NFκB and caspases to exert its functions.[36] Moreover, fucoxanthin could also regulate the mRNA expression of many proteins, such as TNF-α, MCP-1 and SCD1 in cells and animals.[37,38] We further anticipated that fucoxanthin acted on pro-survival and/or pro-apoptotic proteins which are involved in H_2_O_2_-induced oxidative stress. The PI3K/Akt pathway plays an important role in neuronal survival.[39] Phosphorylated Akt further induces the inhibition of GSK3β via phosphorylating its Ser-9 residue.[40] In our study, we found that fucoxanthin significantly protected against H_2_O_2_-induced reduction of pSer473-Akt and pSer9-GSK3β. We also demonstrated that a GSK3β specific inhibitor protected against H_2_O_2_-induced neuronal apoptosis, whereas PI3-K specific inhibitors abolished the neuroprotective effects of fucoxanthin, supporting the role of the PI3-K/Akt/GSK3β cascade in the neuroprotective effects of fucoxanthin. These data were in agreement with a previous study that fucoxanthin can activate the PI3-K/Akt cascade to reduce oxidative stress-induced cell injury in human keratinocytes.[41]

The ERK pathway is one of the key pathways mediating oxidative stress-induced neurotoxicity.[42] We found that fucoxanthin significantly protected against H_2_O_2_-induced enhancement of pERK. Furthermore, an MEK specific inhibitor protected against H_2_O_2_-induced neurotoxicity, suggesting that inhibition of the ERK pathway was also involved in the neuroprotective effects of fucoxanthin. These results were consistent with previous studies that fucoxanthin can inhibit the ERK pathway to ameliorate oxidative stress-induced cell injury in macrophages.[43] Taken together with our previous findings, we concluded that fucoxanthin could both activate the pro-survival PI3K/Akt cascade and inhibit the pro-apoptotic ERK pathway to produce its neuroprotective effects.

In the current study, we only performed cell culture experiments, which is a limitation. In a submitted study [44], we have found that fucoxanthin could prevent β-amyloid (Aβ) oligomer-induced abnormities in learning and memory. Aβ oligomers are widely accepted as main neurotoxins to induce neuronal impairments in Alzheimer’s disease. Aβ oligomers lead to neuronal death mainly via oxidative stress.[45] Therefore, these animal studies further confirmed our current findings that fucoxanthin might produce neuroprotective effects against oxidative stress-induced neurotoxicity.

In summary, we, for the first time, found that fucoxanthin protected against H_2_O_2_-induced apoptosis via concurrently activating the PI3K/Akt cascade and inhibiting the ERK pathway. Our results also provided support for the use of fucoxanthin in the treatment of neurodegenerative disorders caused or characterized by oxidative stress.
